# Correction to: Phase Ib/II Study of Biweekly TAS‐102 in Combination with Bevacizumab for Patients with Metastatic Colorectal Cancer Refractory to Standard Therapies (BiTS Study)

**DOI:** 10.1093/oncolo/oyaf119

**Published:** 2025-05-30

This is a correction to: Hironaga Satake, Takeshi Kato, Koji Oba, Masahito Kotaka, Yoshinori Kagawa, Hisateru Yasui, Masato Nakamura, Takanori Watanabe, Toshihiko Matsumoto, Takayuki Kii, Tetsuji Terazawa, Akitaka Makiyama, Nao Takano, Mitsuru Yokota, Yoshihiro Okita, Koreatsu Matoba, Hiroko Hasegawa, Akihito Tsuji, Yoshito Komatsu, Takayuki Yoshino, Kentaro Yamazaki, Hideyuki Mishima, Eiji Oki, Naoki Nagata, Junichi Sakamoto, Phase Ib/II Study of Biweekly TAS‐102 in Combination with Bevacizumab for Patients with Metastatic Colorectal Cancer Refractory to Standard Therapies (BiTS Study), *The Oncologist*, Volume 25, Issue 12, December 2020, Pages e1855–e1863, https://doi.org/10.1634/theoncologist.2020-0643

In the originally published version of the manuscript, there was an error in the table **PATIENT CHARACTERISTICS**. Under subheading **Detailed Patient Characteristics**, the table lists the *“RAS* status” as follows:

”Wild-type: 25 (56.8%)

Mutant: 19 (43.2%)”

However, the correct values should be transposed:

”Wild-type: 19 (43.2%)

Mutant: 25 (56.8%)”

The labels were reversed due to a transcription error. The mistake does not affect any other part of the paper. This error has been outlined only in this correction notice to preserve the version of record.

